# Perception of stress by medical staff during the first wave of the COVID-19 pandemic in russia

**DOI:** 10.1192/j.eurpsy.2021.751

**Published:** 2021-08-13

**Authors:** V. Barabantschikova, O. Vakhantseva, O. Almazova, L. Shaigerova, R. Shilko

**Affiliations:** Faculty Of Psychology, Lomonosov Moscow State University, Moscow, Russian Federation

**Keywords:** mental health, stress level, COVID-19, health care workers

## Abstract

**Introduction:**

Assessment of mental health in medical staff during the COVID-19 pandemic helps to prevent the delayed negative consequences of work under high risk.

**Objectives:**

The study compares the perceived stress and coping ability in medical staff caring for patients diagnosed with COVID-19 and in professionals, working with other patients.

**Methods:**

Online survey involved 249 doctors and medical staff of a Moscow hospital aged 19 to 60 (80 men and 169 women). 135 people worked directly with COVID-19 patients and 114 specialists did not. The study was conducted with the Perceived Stress Scale (10-items) amidst the first wave of COVID-19 in Russia, in May 2020.

**Results:**

No significant differences were found in the stress levels between the two groups of healthcare providers. On average, the sample revealed an increased level of stress (15.4 with standard values of 12-13). The general indicator of perceived stress (F = 13.471; p <0.001), stress level (F = 12.333; p = 0.001) and stress resistance (F = 6.003; p = 0.015) in women is significantly higher than in men. In addition, women working with COVID-19 patients have a higher level of stress resistance than women working with other patients (F = 3.432; p = 0.045).

**Conclusions:**

The perception of stress by healthcare staff during the COVID-19 pandemic increases independently on whether or not they work with infected patients. Although stress is higher in women, they are better at coping, especially in extreme situations. The reported study was funded by RFBR, project number 20-04-60174.

